# Do Emotional Cues Influence the Performance of Domestic Dogs in an Observational Learning Task?

**DOI:** 10.3389/fpsyg.2021.615074

**Published:** 2021-05-20

**Authors:** Natalia Albuquerque, Carine Savalli, Francisco Cabral, Briseida Resende

**Affiliations:** ^1^Department of Experimental Psychology, Institute of Psychology, University of São Paulo, São Paulo, Brazil; ^2^Department of Public Policies and Collective Health, Federal University of São Paulo, São Paulo, Brazil

**Keywords:** *Canis familiaris*, emotion, social cognition, social information, socially biased learning

## Abstract

Using social information is not indiscriminate and being able to choose what to copy and from whom to copy is critical. Dogs are able to learn socially, to recognize, and respond to dog as well as human emotional expressions, and to make reputation-like inferences based on how people behave towards their owner. Yet, the mechanisms dogs use for obtaining and utilizing social information are still to be fully understood, especially concerning whether emotional cues influence dogs’ social learning. Therefore, our main aim was to test the hypothesis that an emotionally charged (negative, positive, or neutral) interaction with the demonstrator of a “V” detour task prior to testing would affect subjects’ performance, by: (i) changing the value of the information provided by the demonstrator or (ii) changing the valence of the learning environment. Our experimental design consisted of three phases: pre-test (subjects were allowed to solve the task alone); emotional display (dogs watched the unfamiliar human behaving in either a positive, negative or neutral way towards their owner); test (demonstrator showed the task and subjects were allowed to move freely). Only dogs that failed in pre-test were considered for analysis (*n* = 46). We analyzed four dependent variables: *success*, *time to solve the task*, *latency to reach the fence* and *matching* the side of demonstration. For each, we used four models (GEEs and GLMMs) to investigate the effect of (1) demographic factors; (2) experimental design factors (including emotional group); (3) behavior of the dog; and (4) side chosen and matching. All models took into account all trials (random effect included) and the first trials only. Our findings corroborate previous studies of social learning, but present no evidence to sustain our hypothesis. We discuss the possibility of our stimuli not being salient enough in a task that involves highly motivating food and relies on long and highly distracting interval between phases. Nevertheless, these results represent an important contribution to the study of dog behavior and social cognition and pave the way for further investigations.

## Introduction

Social life is advantageous in many ways. For instance, the possibility of an efficient communication between individuals (facilitated and maintained by signaling and perception) enables affiliative and/or cooperative interactions among social animals (Fedurek et al., 2015). Moreover, learning socially provides a flexible way of acquiring information that can reduce the costs often involved in the acquisition of resources and new skills (Zentall, 2006). Learning from others is key for the evolution of social behavior and allows children, as well as other animals, to acquire ecologically relevant information regarding their physical and social environment (Flynn and Whiten, 2013). Therefore, for individuals that live in cohesive groups, visual and acoustic cues, such as pointing, gazing, vocal and facial expressions act as important signals, thus, providing adaptive advantages for assessing and responding to experience without the need for direct interaction (Colbert-White et al., 2018).

Social learning can be defined as “learning facilitated by observation of, or interaction with, another individual or its products” (Heyes, 1994; Hoppitt and Laland, 2013). According to Canteloup et al. (2020), it can lead an organism to behave in a different way after watching another one act in a particular manner. Social learning usually occurs between conspecifics, that are well equipped with repertoires of social learning capacities to deal with the information provided by similar individuals (e.g., Whiten and Mesoudi, 2008; Legare, 2017). However, it often occurs between different species. This is the case for some bird species, as demonstrated by Seppanën and Forsman (2007), who has shown that migratory birds (flycatchers) can learn from resident birds (tits). The flycatchers used the other better-informed birds as a source of information and acquired their nest site preferences. Another example that has caught the attention of researchers involves dogs and humans. In fact, when looking at dogs, the investigation of interspecific processes is critical, due to their close and intimate relationship with people (e.g., Guillo and Claidière, 2020).

Social learning can ascribe a diversity of processes, such as social, local and stimulus enhancement, social facilitation, perceptual biases and others (Heyes and Galef, 1996; Hoppitt and Laland, 2013; Eschar et al., 2016; Lind et al., 2019). Taken together, these processes composite what authors such as Fragaszy and Visalberghi (2004) call socially biased learning. Goal-directed actions contain two main sources of information that can be gathered through observation: (i) the movement and (ii) the consequence. Imitation occurs when an observer learns specific aspects of another’s actions, whilst emulation occurs when an observer learns about the effects of one’s actions and copies the outcome (Heyes, 1993; Flynn and Whiten, 2013). However, the factors influencing whether what learning processes are in place for different animals in different contexts is an intriguing question (Fugazza et al., 2019). In several cases, a given behavior may have different weights depending on characteristics of the individual who is being observed (Canteloup et al., 2020). In fact, social learning strategies may differ greatly in form. Laland (2004), discusses the importance of assessing the nature of the strategies used during social learning, especially in term of the contexts where it occurs. According to Coelho et al. (2015), complementary to comprehending the underlying mechanisms is to address the questions “when to copy,” “what to copy” and “whom to copy.”

Such aspects are only actually beneficial if one is sensitive to and can remember how others have acted in past interactions. Humans, for example, since their first year of life tend to approach more individuals who have acted positively towards others and to avoid more individuals who have acted negatively, even though during development these preferences may not be as straight forward (Hamlin et al., 2011). In fact, the capacity to acquire new skills and knowledge by observing others is so critical to the development of humans that children as young as 2 years old will even imitate irrelevant actions (overimmitate) that they know are unnecessary to achieving an instrumental goal (Legare and Nielsen, 2015). However, the ability to imitate social partners is not restricted to humans. For instance, Caldwell and Whiten (2004) showed that common marmosets manipulate and interact more with an artificial fruit after a trained conspecific has given a full demonstration of how to open the artificial foraging task, compared to partial or no demonstration conditions, and Huber et al. (2020) have recently shown that dogs selectively imitate their caregivers, but not strangers.

The ability to discriminate individuals by their social role is critical for people. For instance, humans must assess the motivations, intentions and emotional reactions of others to make accurate decisions of who is and who is not an appropriate partner. In fact, this ability is found in human beings from very young ages, with preverbal infants already showing to evaluate others based on their behavior in different social contexts (Hamlin et al., 2007). The information individuals acquire is crucial to channel their decision-making (McFarland et al., 2013) and positive or negative third-party interactions might change the value of another individual as, for example, a social partner and, in a social learning context, someone that must be copied.

In addition to interacting with conspecifics, humans establish long lasting, dynamic, complex, and mutually advantageous relationships with domestic dogs (Albuquerque and Savalli, 2017; Savalli et al., 2019). They have co-existed for at least 10,000 years with genetic evidence suggesting more than 20,000 years of divergence between the ancestor of the modern gray wolf and the ancestor of the domestic dog (Skoglund et al., 2015; Pendleton et al., 2018). During this co-shared evolutionary history, dogs are believed to have developed cognitive capacities to better interact with humans (e.g., Nagasawa et al., 2015). For instance, they are very sensitive to human communicative cues (Hare et al., 2002; Reid, 2009; Dahas et al., 2013; Ford et al., 2019), in addition to producing signals to communicate with people (Savalli et al., 2014, 2016), and having the capacity to process, recognize and respond to human emotional expressions (Albuquerque et al., 2016, 2018; Somppi et al., 2016; Albuquerque, 2017; Kujala, 2018). Moreover, these animals can obtain information from humans about a novel object or an uncertain situation by observing their reactions towards the stimulus (Merola et al., 2012).

Dogs are sensitive to the behavior of others and use the social information they obtain from direct and indirect social interactions to solve problems (e.g., Topál et al., 2006; Range et al., 2007), both from conspecifics (Scandurra et al., 2016) and from humans (Pongrácz et al., 2001, 2003). In the early 2000’s, Pongrácz and colleagues investigated whether dogs could learn socially. In 2001, they used a detour task, where dogs should reach a desired object positioned on one of the sides of a “V” shaped fence. Dogs showed a low rate of success when the movement was inwards. However, when a person was included as a demonstrator to show how to solve the problem, subjects decreased the time they took to solve the task and became proficient at the task in both directions.

On the other hand, dogs act differentially towards people even after a brief exposure to them. This happens because, like humans, domestic dogs can assign reputation-like statuses to other individuals that will be taken into account when choosing with whom to interact (Kundey et al., 2011). These assessments can occur directly but also indirectly, through the observation of third-party interactions. In fact, dogs can discriminate a generous from a selfish food-sharing person from observing the interaction between two people (e.g., Marshall-Pescini et al., 2011). Research has shown this discriminatory capacity may be more related to the presence of food than to the actual evaluation of the social role of each person (Piotti et al., 2017), however, Chijiiwa et al. (2015) controlled for these possible confounding effects and showed that dogs are indeed capable of assessing third-party interactions, discriminate social roles (e.g., helper vs non helper) and avoid the person who has behaved negatively towards their owner. Carballo et al. (2016, 2017) discuss that both the domestication process and the amount of experience dogs have with people influence these abilities.

Even though there is an increasing body of literature on dogs’ abilities to learn from observation, to make reputation-like inferences and to recognize emotional expressions of humans, little is known about the influence affective cues and/or affective impressions pose on the capacity to obtain context-relevant information and to learn socially. In this study, we used the “V” detour task and a demonstrator with potentially different social weights, which were determined by her immediately prior interaction with the dog’s owner in the presence of the dog. The demonstrator of the task, who was completely unfamiliar to the subject at that time, acted in either a positive, a negative or a neutral way towards the owner during a conversation. We combined adaptations of the classical “V” detour setting (Pongrácz et al., 2001, 2003) with a very thorough behavior codification of 46 analyzed subjects to better comprehend the nuances involved in domestic dogs’ social learning, including what mechanisms are used in this sort of observational learning task. The experimental design consisted of three phases: pre-test (subjects were allowed to solve the task alone); emotional display (dogs watched the unfamiliar human behaving emotionally towards their owner); test (demonstrator showed the task and subjects were allowed to move freely).

We tested the hypothesis that the observation of third-party affective interactions between owner and demonstrator of the social learning test can either facilitate or impair subjects’ learning (measured by completion of the task) depending on the valence of the interaction. We predicted the emotional displays by the unfamiliar person would affect the context in two ways: (i) changing the valence of the environment/situation (i.e., positive interaction would create a positive environment and could facilitate learning) and/or (ii) changing the value of the demonstration and, consequently, of the information regarding the detour (i.e., positive demonstrators would be seen by the dogs as providers of higher quality or more relevant information and negative demonstrators would be seen as having less relevant or lower quality information). Therefore, we expected dogs in the positive group to show higher rates of success, lower time to solve the task, higher matching (i.e., choosing the same side as demonstrator) and lower latencies, followed by dogs in the neutral group and, last, by dogs in the negative group. Moreover, we looked at other behaviors (looking at owner, standing still next to the owner, persistence, distraction, and time spent looking at demonstrator during demonstration of the task) in relation to the emotional group subjects had been assigned to in order to have a fuller understanding of the phenomena.

## Materials and Methods

### Ethics Statement

All experimental procedures complied with the ethical guidance for the use of animals produced by the International Society for Applied Ethology. The study was approved by the Animal Ethics Committee of the University of São Paulo (USP) (CEUA n^*o*^ 1567110915) and did not involve any invasive measurements or caused any psychological discomfort to the subjects. The behavior of the dogs were monitored throughout the entire experimental session and in case of signs of distress, testing was terminated. Prior to the start of the experimental session, the owner was informed about the general lines of the study and signed a consent form.

### Subjects

We tested a total of 52 healthy well socialized family adult dogs of various breeds. However, six dogs had to be excluded from the analyses due to having had success in the pre-test (see below for detailed information). Therefore, we analyzed the behavior of 46 dogs (30 females and 16 males), aged between 2 and 10 years old (Table 1). Participation was voluntary. The study was advertized in social media platforms, as well as in veterinary clinics, pet stores, etc., and owners voluntarily applied for participation. Suitable dogs, i.e., dogs that were used to be in unfamiliar places and to interact with unfamiliar people, were recruited after a screening process that consisted in a written semi-structured interview filled in by the owners prior to the experiment day.

**TABLE 1:** Information of the sample of dogs analyzed.

Dog	Emotional group	Side of 1st demonstration	Sex	Age (months)	Breed
1	Negative	Left	Male	48	Mongrel
2	Negative	Right	Female	48	Mongrel
3	Positive	Left	Male	48	Mongrel
4	Negative	Right	Male	30	Mongrel
5	Positive	Right	Female	96	Mongrel
6	Positive	Right	Female	84	Border Collie
7	Negative	Left	Female	15	Mongrel
8	Negative	Right	Female	84	Lhasa Apso
9	Positive	Left	Female	60	Havanese
10	Negative	Right	Male	69	Shetland Sheepdog
11	Positive	Left	Male	24	Shetland Sheepdog
12	Positive	Right	Female	48	Mongrel
13	Negative	Left	Female	72	Golden Retriever
14	Negative	Left	Male	72	Mongrel
15	Positive	Right	Female	72	Rottweiler
16	Negative	Left	Male	60	Mongrel
17	Positive	Right	Female	96	Mongrel
18	Negative	Left	Female	60	Mongrel
19	Positive	Right	Female	60	Pitbull
20	Negative	Left	Male	36	West Terrier
21	Positive	Left	Male	15	French Bulldog
22	Neutral	Left	Male	36	Mongrel
23	Negative	Left	Female	36	Mongrel
24	Neutral	Left	Male	24	Poodle
25	Neutral	Right	Male	48	Yorkshire
26	Neutral	Left	Female	102	Mongrel
27	Neutral	Left	Female	30	Pug
28	Neutral	Left	Female	24	Mongrel
29	Positive	Right	Female	60	Australian Cattle Dog
30	Neutral	Right	Female	96	Daschund
31	Positive	Right	Female	60	Rottweiler
32	Neutral	Right	Female	57	Labrador
33	Neutral	Left	Male	96	Labrador
34	Neutral	Left	Female	36	Labrador
35	Neutral	Right	Female	20	Mongrel
36	Negative	Right	Female	60	Schnauzer
37	Neutral	Left	Female	16	Pitbull
38	Negative	Left	Female	36	French Bulldog
39	Neutral	Right	Female	48	English Cocker Spaniel
40	Negative	Right	Male	123	Sheepdog
41	Neutral	Left	Male	21	Mongrel
42	Neutral	Right	Male	42	Mongrel
43	Positive	Left	Female	96	Yorkshire
44	Neutral	Right	Female	19	Pinscher
45	Negative	Right	Female	84	Mongrel
46	Neutral	Right	Female	114	Poodle

### Experimental Procedures

**FIGURE 1:**
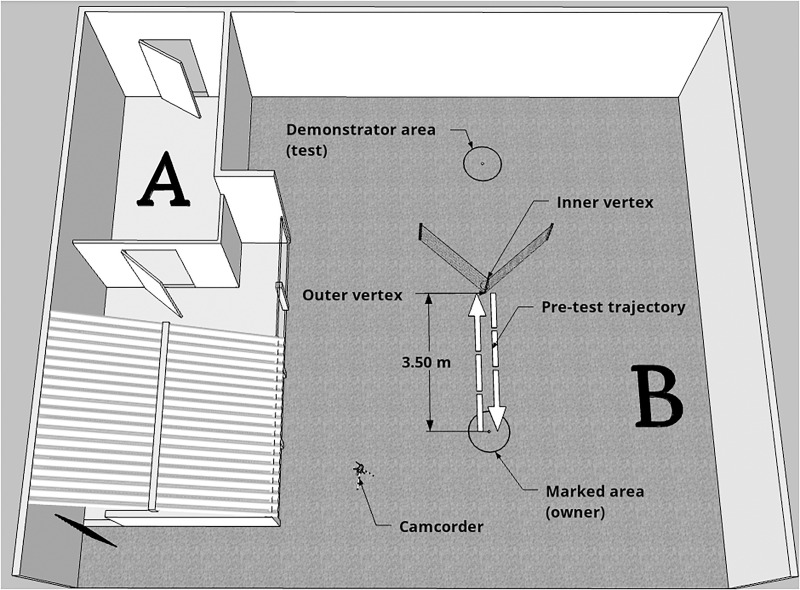
Schematic representation of the experimental area. The emotional display phase happened in Space A, whereas pre-test and test occurred in Space B. Dashed arrows represent the trajectory taken by the owner and dog during pre-test (with the aim of showing the dog the baited bowl placed in the inner vertex of the fence).

Data collection was conducted at the External Ethology Laboratory of the Institute of Psychology of USP, during a period of 12 months. The experimental environment consisted of two open-air spaces (Figure 1). In space A, the emotional demonstration phase was conducted, whereas pre-test and test were conducted in space B. Experimenter 1 (E1; demonstrator), who was always the same person between dogs, stayed hidden until the experiment started in order to guarantee the subject had absolutely no experience with her. Experimenter 2 (E2), who could vary between testing days, was trained to meet the owner and the dog outside the laboratory and provide the instructions of the experiment. Before the beginning of the experiment, dogs were given a period of free time (approxixmately 10 min) to explore the environment, so they would lose interest in the area, and get habituated and comfortable. From the moment E2 and owner understood the dog was habituated, the experiment was started.

**FIGURE 2:**
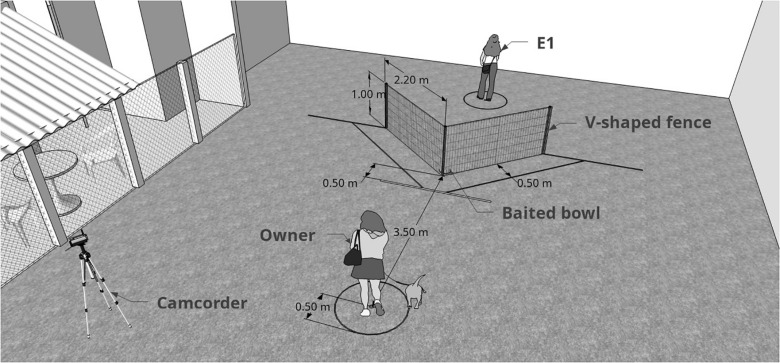
Graphic representation of the moment when the subject was about to be released and start the test. E1 (demonstrator) had already baited the bowl and was distant from the fence, facing the opposite side). The difference between pre-test and test is the presence of the demonstrator. Measurements shown are those from the real experiment.

Subjects were tested in an adapted form of the classic “V” detour task (Pongrácz et al., 2001, 2003), where dogs are placed in front of a V-shaped fence with a baited bowl in its inner vertex (Figure 2) and witness a demonstration from a knowledged individual (human) of how to access the bowl. The experiment was divided into three distinct experimental phases: pre-test (subjects allowed to solve the task alone); emotional display (dogs watched the unfamiliar experimenter behaving in either a positive, negative or neutral way towards the owner); test (experimenter demonstrated the task and subjects were allowed to solve it). Each dog was tested in 10 similar trials that only differed on side of demonstration (left or right), which was counterbalanced along trials. First side of demonstration was randomized between subjects. Only dogs that were not successful in pre-test were considered for analysis (*n* = 46). E1 acted as the person who interacted emotionally (positive manner, negative manner, or neutral manner) towards the owner and as the demonstrator of the social learning task. E1 only interacted with the dog after the experiment was finalized.

All experimenters were trained before the start of the experiment. The emotional displays (see below) were extensively trained with E1 until the sentence pronunciation and emotional cues were consistent and robust.

### Pre-test

Immediately after habituation, E2 instructed the owner about pre-test, which would be a trial without the demonstrator to see whether the dog could solve the detour task alone: if they could, that would mean that no social learning would occur. Therefore, performance on the pre-test served as a criterion to include – or not – the dogs in the analysis.

As soon as E2, owner and dog entered space B (see Figure 1), E2 took them to the previously marked area (3.5 m away from the vertex of the fence) where owner and dog would stand during the demonstration of the task in the test phase, and instructed the owner to walk towards the outer vertex of the fence in a straight line in order to let the dog see the baited bowl. The dog was on the leash at this point. The bowl, which was on the inner vertex of the fence, was not reachable, but was visible and the dogs could smell the food inside. As soon as the dog appeared interested to get the food, E2 instructed the owner to come back to the marked area. Once owner and dog were set, E2 gave the command and the owner unclipped the dog’s leash. At this point, the dog could move completely free and the owner stood still, neutral and did not interact with the dog by any means. Whether the dog could reach the baited bowl in the inner vertex of the fence or not was recorded and used to select the subjects that would be considered in the data analysis. Each subject was allowed one pre-test that lasted 15 s. After this time, the owner was asked to retrieve the dog and accompany E2 outside the experimental area.

### Emotional Display Phase

**FIGURE 3:**
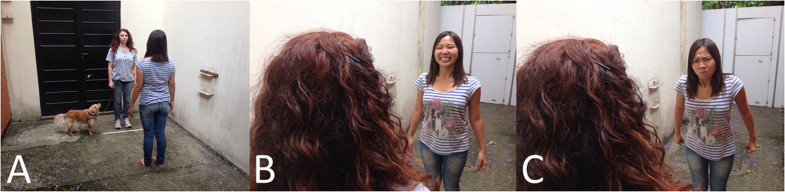
Example of the emotional display phase. **(A)** Shows where the owner was positioned, **(B)** depicts a positive emotional display, and **(C)** depicts a negative emotional display.

In the meanwhile, E1 entered the experimental area and stayed between space A and B. This particular area had an opaque door separating space A, which E1 closed after her entrance. That way, dog and owner could return to space A without seeing E1. The owner was instructed to stay still, looking at the demonstrator and to keep a neutral facial expression and body position. In addition, they should never interact with the dog, who was on the leash but could move. As soon as the owner and dog were positioned in the pre-determined area (see Figure 3 for an example), E1 entered space A making eye-contact solely with the owner. The display of the emotional stimulus occurred only once for each subject and was directed to the person. The stimulus could be either positive, negative or neutral and consisted of the pronunciation of the sentence “You know what I mean,” in English, in order to avoid any familiarity or habituation effect with any of the words used by the Brazilian owners with their dogs. The sentence was repeated three times, each with the intonation correspondent to the designated valence, together with the congruent body and facial emotional cues (Figure 3). Dogs were previously allocated to one of the three groups: positive, neutral or negative in a randomorder.

### Test

After the emotional interaction, E1 left space A through the opaque door (still in the character, i.e., positive, neutral or negative) and E2 entered space A to continue guiding the owner. While E2 was providing the instructions regarding the next phase of the test, E1 positioned herself at the outer vertex of the fence, standing still, with a neutral face and treat bag clipped to her belt. Once set, owner, dog and E2 entered space B and positioned themselves in the pre-determined area (the same as in pre-test). At this stage, the owner stood still with a neutral face and body position throughout the entire test, never interacted with the dog regardless of their behavior and looked straight ahead. Subjects who had their owner interfering in any way were excluded from analyses. Soon after everyone was set in place, E1 clapped her hands three times to gain the subject’s attention to start the silent demonstration of the detour task. Once eye contact was established, she initiated the detour using one side of the fence (previously determined and randomized between subjects). When she reached the bowl, she leaned down, put treats (small pieces of fresh cheese and sausage) from her bag inside and then moved away from the set up to the previously determined area (demonstrator area, see Figure 1). From this point until the end of the trial, she kept her back turned to the setting, keeping a neutral manner, and did not move or looked at the dog (see Figure 2). E2 gave the owner the command to unclip the leash. The dog was then free to move freely for 30 s. Success was considered when the subject reached the bowl within this period. If the dog completed the task before 30 s, E2 asked the owner to bring back the dog after letting them eat the food. Dog’s behavior was recorded by a digital video camera for *post hoc* analysis. After this period, E2 asked the owner to retrieve the dog and come back to the marked position. Each dog was presented to 10 trials, alternating side of demonstration, which consisted of repetitions of the above mentioned. Before pre-test and test, to avoid potential olfactory cues, the demonstrator walked around the fence ten times (five deviations to the left side and five deviations to the right side).

### Data Coding and Statistical Analysis

During testing, E2 manipulated a chronometer, used to control for testing time, and a paper sheet, where she/he marked whether the dog had success, the time to solution and the chosen side in each trial. However, in order to generate more robust data and collect more detailed information, we analyzed the videos with the software Solomon Coder Beta^1^ using real speed and frame-by-frame coding, looking at the mentioned variables and also another complementary behaviors.

A second naïve person coded a random sample of 25% of the videos. Both coders were blind to the test group of all dogs and Kendall’s concordance coefficient was calculated: time watching the demonstrator during test (*W* = 0.97), looking at the owner (*W* = 0.74), looking at the bowl (*W* = 0.83), latency to reach the fence (*W* = 0.93), sniff the fence (*W* = 0.67), and, sniff the environment *W* = 0.76).

We analyzed four main dependent variables: *success* in solving the task, *time to solve the task* for those who solved it, *latency to reach the fence* (defined as the time the subject took to arrive at the outer vertex of the fence) and *matching* of side, i.e., when the dog chose the same side as the demonstration. Matching and latency to reach the fence were also used as independent variables for verifying potential effects on the other response variables. Success in solving the task and matching, which were binary responses, were analyzed using a binomial generalized linear mixed model (GLMMs) with a logit link function and dog as a random effect when considering all trials. A logistic regression was used when considering the first trial only. For these models, estimates were presented as odds ratio (OR). Time to solve the task and latency to reach the fence were analyzed using a linear mixed model (LMMs) with dog as a random effect when considering all trials. Regression models were run when considering the first trial only.

For each dependent variable, we used four models: (1) effect of demographic factors (sex, age and breed); (2) effect of experimental design factors (emotional stimulus, trial segment and side of demonstration); (3) effect of behavior of the dog (time spent watching the demonstrator during the test, whether the dog looked at the owner for some time (yes/no), whether the dog stood still next to the owner for some time (yes/no), persistence score (defined below), distraction score (defined below), duration until reaching the fence (latency to reach the fence) – except when this variable was the response; and (4) effect of side chosen by the dog and matching – except when this variable was the response. A “side chosen” was considered as soon as the dog had passed the outer vertex of the fence in one of the two possible directions: left or right. This measure has been chosen to verify any side biases and to test for how dogs match their behavior to that of the demonstrator. All models were conducted using all trials (random effect included) and the first trials only. For models with all trials, segments of trials were used as factor to evaluate the learning effect. To do so, trials were divided into three categories (initial: the first three trials, middle: the four middle trials, and, final: the final three trials). For analysing the behavior of dogs, we used what we called “persistence score” and “distraction score.” Persistence score was defined as the number of behaviors presented among the list of actions: digging in front of the fence, touching the fence, sniffing the fence, and looking at the bowl. Distraction score was defined as the number of behaviors presented among the list of actions: urinating, defecating, seeking for noises, sniffing the environment, and digging the ground. We chose to divide the analysis in different models because we considered that each group of variables intended to evaluate a different facet of the phenomenon studied.

Prior to running the models, in order to evaluate the homogeneity of groups of dogs distributed among emotional stimulus (positive, negative or neutral) with respect to sex, breed and the side of first demonstration, we used the Chi-square test. In addition, with respect to the dogs’ age, we used the one-way Analysis of Variance.

All behaviors were coded from the records of the test phase, mostly from when the dog was free to move in the setting. In addition, we analyzed the behavior “time spent looking at demonstrator” during the demonstration of the task by E1, which was not accounted for when dogs had been unclipped, because at that point, E1 was away from the fence, facing backwards and standing completely still and dogs showed very little or no interest in her. Data was collected for the entire test, however the dogs almost never looked at the demonstrator when they were free to move, meaning that we had too many zeros and, thus, could only analyse data from when the dogs were still on the leash. On the other hand, the other behaviors have not been accounted for during demonstration, because at that point, dogs were on the leash, next to the owner, and mostly visually following the demonstrator.

Models residuals and fitting were checked. The software SAS University Edition was used for all statistical analyses. We used a 5% significance threshold with Bonferroni correction for each model for interpretation of the results. The significance level for models 1–4, were, respectively, 1.7, 1.7, 0.8, and 2.5%. The ethogram used for behavioral codification is included in supplementary materials as Supplementary Table 1.

## Results

From a total sample of 52 tested dogs, the six dogs who passed pre-test had to be excluded from analysis. Thus, the sample analyzed consisted of 46 subjects (30 females and 16 males) with an average age of 52.1 months (standard deviation = 28.8), of various breeds (27 purebred and 19 mongrels). From the sample, 33 solved the task in the first test trial. Trials altogether were a total of 460 (two missing trials), from which 150 ended up in success. Regarding the emotional display groups, 16 dogs were exposed to the negative emotional stimulus, 16 to the neutral stimulus and 13 to the positive stimulus.

Since a few dogs were excluded, we analyzed whether the distribution among emotional groups (positive, negative, and neutral) was balanced for demographic variables and side of first demonstration. We found no bias for sex (male vs female), breed (purebred vs mongrel), age and side (left or right).

In model 1, in which we took into account the demographic characteristics of the subjects (i.e., sex, age, and breed), we found only an effect of age on matching, when looking at all trials (*F*_1,179_ = 7.53, *p* = 0.0067). The greater the age the smaller the odds of matching (Odds Ratio = 0.965, CI95% = [0.941;0.990]). No effect was found for success, time to solve the task and latency to reach the fence (Table 2).

**TABLE 2:** Summary of the results of the models (statistic and *p*-value).

	Response variables							
	Success in solving the task		Time to solve the task		Latency to the fence		Matching	
Models	All trials	1st trial	All trials	1st trial	All trials	1st trial	All trials	1st trial
**Model 1 – Effect of demographic factors**								
Sex	*F*_(1,412)_ = 0.24 *p* = 0.6210	$X_{1}^{2}$ = 0.30 *p* = 0.5829	*F*_(1,123)_ = 1.63 *p* = 0.2041	*F*_(1,9)_ = 0.34 *p* = 0.5755	*F*_(1,412)_ = 1.77 *p* = 0.1847	*F*_(1,42)_ = 0.01 *p* = 0.9066	*F*_(1,179)_ = 0.63 *p* = 0.4276	$X_{1}^{2}$ = 0.51 *p* = 0.4749
Age	*F*_(1,412)_ = 1.67 *p* = 0.5703	$X_{1}^{2}$ = 1.74 *p* = 0.1873	*F*_(__1,123)_ = 0.03 *p* = 0.8658	*F*_(__1,9)_ = 0.73 *p* = 0.4137	*F*_(__1,412)_ = 0.31 *p* = 0.5750	*F*_(__1,42)_ = 0.62 *p* = 0.4348	*F*_(__1,179)_ = 7.53 ***p* = 0.0067**	$X_{1}^{2}$ = 3.87 *p* = 0.0493
Breed	*F*_(__1,412)_ = 0.32 *p* = 0.5702	$X_{1}^{2}$ = 0.11 *p* = 0.7418	*F*_(__1,123)_ = 0.18 *p* = 0.6753	*F*_(__1,9)_ = 0.04 *p* = 0.8444	*F*_(__1,412)_ = 0.20 *p* = 0.6541	*F*_(__1,42)_ = 0.21 *p* = 0.6465	*F*_(__1,179)_ = 1.78 *p* = 0.1843	$X_{1}^{2}$ = 0.46 *p* = 0.4964
**Model 2 – Effect of experimental design**								
Emotional stimulus	*F*_(__2,410)_ = 0.40 *p* = 0.6675	$X_{1}^{2}$ = 0.64 *p* = 0.7275	*F*_(__2,121)_ = 1.50 *p* = 0.2277	*F*_(__2,9)_ = 0.88 *p* = 0.4474	*F*_(__1,410)_ = 0.25 *p* = 0.7796	*F*_(__2,42)_ = 1.71 *p* = 0.1933	*F*_(__2,177)_ = 1.41 *p* = 0.2466	$X_{2}^{2}$ = 0.21 *p* = 0.9016
Trial segment	*F*_(__2,410)_ = 6.28 ***p* = 0.0021**	–	*F*_(__2,121)_ = 8.36 ***p* = 0.0004**	–	*F*_(__1,410)_ = 6.72 ***p* = 0.0013**	–	*F*_(__2,177)_ = 1.39 *p* = 0.2525	–
Side of demostrantion	*F*_(__1,410)_ = 0.06 *p* = 0.8072	$X_{1}^{2}$ = 0.08 *p* = 0.7799	*F*_(__1,121)_ = 0.52 *p* = 0.4714	*F*_(__1,9)_ = 2.94 *p* = 0.1208	*F*_(__1,410)_ = 2.74 *p* = 0.0989	*F*_(__1,42)_ = 1.55 *p* = 0.2193	*F*_(__1,177)_ = 0.87 *p* = 0.3525	$X_{1}^{2}$ = 0.36 *p* = 0.5497
**Model 3 – Effect of behaviour of the dog**								
Time watching demonstrator (test)	*F*_(__1,406)_ = 0.08 *p* = 0.7763	$X_{1}^{2}$ = 0.39 *p* = 0.5306	*F*_(__1,117)_ = 0.33 *p* = 0.5671	*F*_(__1,6)_ = 0.17 *p* = 0.6944	*F*_(__1,407)_ = 15.37 ***p* = 0.0001**	*F*_(__1,40)_ = 11.89 ***p* = 0.0013**	*F*_(__1,173)_ = 0.29 *p* = 0.5900	$X_{1}^{2}$ = 2.43 *p* = 0.1186
Whether the dog looked at the owner	*F*_(__1,406)_ = 22.68 ***p* < 0.0001**	$X_{1}^{2}$ = 2.49 *p* = 0.1148	*F*_(__1,117)_ = 0.70 *p* = 0.4051	*F*_(__1,6)_ = 0.14 *p* = 0.7250	*F*_(__1,407)_ = 0.08 *p* = 0.7712	*F*_(__1,40)_ = 0.85 *p* = 0.3625	*F*_(__1,173)_ = 0.60 *p* = 0.4387	$X_{1}^{2}$ = 0.002 *p* = 0.9640
Whether the dog stood still next to the owner	*F*_(__1,406)_ = 0.61 *p* = 0.4352	$X_{1}^{2}$ = 0.09 *p* = 0.7674	*F*_(__1,117)_ = 1.76 *p* = 0.1868	*F*_(__1,6)_ = 0.38 *p* = 0.5598	*F*_(__1,407)_ = 89.13 ***p* < 0.0001**	*F*_(__1,40)_ = 16.48 ***p* = 0.0002**	*F*_(__1,173)_ = 1.24 *p* = 0.2674	$X_{1}^{2}$ = 0.57 *p* = 0.4510
Persistence score	*F*_(__1,406)_ = 2.72 *p* = 0.0997	$X_{1}^{2}$ = 1.00 *p* = 0.3159	*F*_(__1,117)_ = 42.52 ***p* < 0.0001**	*F*_(__1,6)_ = 5.10 *p* = 0.0646	*F*_(__1,407)_ = 258.75 ***p* < 0.0001**	*F*_(__1,40)_ = 19.01 ***p* < 0.0001**	*F*_(__1,173)_ = 0.00 *p* = 0.9460	$X_{1}^{2}$ = 0.11 *p* = 0.7370
Distraction score	*F*_(__1,406)_ = 2,51 *p* = 0.1141	$X_{1}^{2}$ = 0.17 *p* = 0.6771	*F*_(__1,117)_ = 11.98 ***p* = 0.0007**	*F*_(__1,6)_ = 0.06 *p* = 0.8201	*F*_(__1,407)_ = 9.00 ***p* = 0.0029**	*F*_(__1,40)_ = 0.14 *p* = 0.7132	*F*_(__1,173)_ = 0.90 *p* = 0.3445	$X_{1}^{2}$ = 0.67 *p* = 0.4116
Latency to reach the fence	*F*_(__1,406)_ = 31.66 ***p* < 0.0001**	$X_{1}^{2}$ = 2.66 *p* = 0.1028	*F*_(__1,117)_ = 59.23 ***p* < 0.0001**	*F*_(__1,6)_ = 7.90 *p* = 0.0307	–	–	*F*_(__1,173)_ = 0.50 *p* = 0.4802	$X_{1}^{2}$ = 1.72 *p* = 0.1892
**Model 4 – Effect of choice**								
Side chosen by the dog	*F*_(__1,177)_ = 3.94 *p* = 0.0488	$X_{1}^{2}$ = 0.30 *p* = 0.5817	*F*_(__1,121)_ = 0.88 *p* = 0.3510	*F*_(__1,10)_ = 0.27 *p* = 0.6154	*F*_(__1,177)_ = 0.63 *p* = 0.4279	*F*_(__1,20)_ = 2.46 *p* = 0.1323	*F*_(__1,178)_ = 2.23 *p* = 0.1373	$X_{1}^{2}$ = 0.006 *p* = 0.9402
Matching	*F*_(__1,177)_ = 0.66 *p* = 0.4187	$X_{1}^{2}$ = 0.005 *p* = 0.9468	*F*_(__1,121)_ = 0.92 *p* = 0.3403	*F*_(__1,10)_ = 0.39 *p* = 0.5481	*F*_(__1,177)_ = 1.39 *p* = 0.2394	*F*_(__1,20)_ = 0.62 *p* = 0.44090	–	–

*For each dependent variable (success, time to solve the task, latency to reach the fence and matching) there are models for all trials and for the first trial. Due to correction for multiple comparisons (Bonferroni approach) the significance level adopted for the models 1–4, were, respectively, 1.7, 1.7, 0.8, and 2.5%).*

**FIGURE 4:**
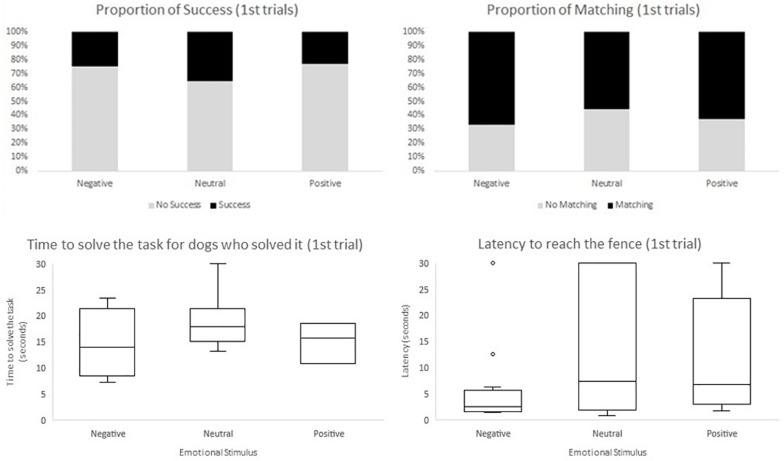
Descriptives of the four dependent variables accounting the first trials, within each emotional group (negative, neutral, and positive). Shown as the proportion of success, the proportion of matching the side of demonstration and the box and whisker plot of time to solve the task and latency to reach the fence.

Regarding the second model, in which we included the experimental design aspects (i.e., emotional stimulus received in the emotional display phase, trial segment, and side of demonstration), we only found a significant effect for trial segment. In the final segment of trials the odds of success were greater when compared to the initial segment (*F*_1,410_ = 5.33, *p* = 0.0215; OR = 1.999, CI95% = [1.108;3.605]), and to the middle segment (*F*_1,410_ = 12.31, *p* = 0.0005; OR = 2.7234, CI95% = [1.554;4.774]). Moreover, time to solve the task (measured in seconds), when there was success, decreased across segment of trials (*F*_2,121_ = 8.36, *p* = 0.0004); initial estimate: 19.679, CI95% = [17.368;21.989]; middle estimate: 18.021, CI95% = [15.775;20.267]; final estimate: 14.964, CI95% = [12.733;17.196]). Initial and final segment were significantly different regarding the time to solve the task (*F*_1,121_ = 15.88, *p* = 0.0001), as well as the middle and final segment (*F*_1,121_ = 7.43, *p* = 0.0074). On the other hand, latency to reach the fence was significantly lower in the initial trials when compared to the middle and final trials (*F*_2,410_ = 6.72, *p* = 0.0013, initial estimate: 11.915, CI95% = [8.918;14.912]; middle estimate: 14.022, CI95% = [11.115;16.929]; final estimate: 15.773, CI95% = [12.770;18.775]). Initial and middle segments were significantly different regarding latency to reach the fence (*F*_1,410_ = 4.60, *p* = 0.0325), as well as the initial and final segment of trials (*F*_1,410_ = 3.15, *p* = 0.0003). No aspect of the experimental design influenced the odds of matching (Table 2). Valence of the emotional display phase (neutral, positive, or negative) and side of demonstration had no effect on the dependent variables considered (Figure 4).

For the third model, we investigated the effect of the behavior of the dog measured by (i) time watching the demonstrator during demonstration of the task, (ii) whether dogs looked at the owner, (iii) whether dogs stood still besides the owner, (iv) persistence score (e.g. not making the detour), (v) distraction score, and (vi) latency to reach the fence. Considering all trials, the odds of success in the task were significantly smaller when the dog looked at the owner at some point (*F*_1,406_ = 22.68, *p* < 0.0001, OR = 0.031, CI95% = [0.008;0.131]). Moreover the greater the latency to reach the fence the smaller the odds to succeed (*F*_1,406_ = 31.66, *p* < 0.0001, OR = 0.874, CI95% = [0.833;0.916]). All other behaviors did not influence the odds of success in the task. When considering only the first trial, no effect on success was found (Table 2). For time to solve the task, we found an effect of persistence (*F*_1,117_ = 42.52 *p* < 0.0001), distraction (*F*_1,117_ = 11.98 *p* = 0.0007) and latency to reach the fence (*F*_1,117_ = 59.23 *p* < 0.0001): time to solve the task increased when these behaviors increased (see Figure 4). When considering only the first trial, for latency to reach the fence, we found an effect of time watching the demonstrator during demonstration of the test (*F*_1,407_ = 15.37 *p* = 0.0001), persistence score (*F*_1,407_ = 258.75 *p* < 0.0001) and distraction score (*F*_1,407_ = 9.00 *p* = 0.0029), all factors were inversely related to latency to reach the fence. Contrarily, latency to reach the fence was greater for dogs that stood still next to the owner (estimate = 19.36, CI95% = [17.79; 20.92]) than for those that did not (estimate = 11.11, CI95% = [9.78;12.44]). These results were also observed when considering only first trials, except for distraction (Table 2). Finally, no significant effect of dogs’ behaviors was found for matching (Table 2).

Lastly, model 4 investigated whether the side dogs chose and matching were related to having success in the task, time to solve the task and latency to reach the fence. Considering all trials, we found no effect regardless the variable.

## Discussion

Our results corroborate Pongrácz et al. (2001, 2003) findings regarding dogs’ capacity to solve the detour task after witnessing the demonstration of the test by a knowledged individual. Pongrácz et al. (2001) observed that dogs alone could not solve a V-shaped detour task from the outside inwards. Therefore, they used a person to demonstrate how to solve the problem and showed that dogs learned from observing the demonstrator how to make the detour. In 2003, the research group used a similar task with a fence that had two open doors, one on each side, which allowed dogs to move through the fence in a faster and easier path to access the food. Pongracz and colleagues found that dogs could imitate humans and would prioritize the demonstrator’s cues instead of their own experience. Even though they could use the doors, dogs made the detour after watching the demonstration. Dogs that did not see a demonstrator used the doors.

In our study, dogs’ success increased as trial segment increased and the time dogs took to solve the task decreased along attempts, which indicates a learning effect across trial segments. Furthermore, when looking at latency to reach the fence, we found that subjects took less time to reach the fence in the initial trials. This last result can be explained by either (i) greater motivation in the beginning; (ii) loss of interest in the task with time and repetition; or (iii) tiredness. A possible explanation could be that dogs needed less time to move as they became proficient in solving the task. However, we found that success was lower when latency to reach the fence was higher, meaning that this latter explanation is unlikely true and some other mechanisms must be in place.

Our results showed that age was the only factor to influence the variable matching (i.e., dogs choosing the same side as the demonstrator). For the analysis of all trials, the only significant effect found was that older dogs were less likely to match their choice with the behavior of the human demonstrator. Even when running a model with the variables “side chosen by dog” and “matching” as independent variables to investigate their potential effect on subjects’ success, time to solve the task, and latency to reach the fence, no significant results were found for matching.

Our findings show that dogs were learning along the trials and were performing better and faster in the test with experience. They also show that dogs were not able to solve the task before the inclusion of a human demonstrator, corroborating previous studies and validating the task used in terms of social learning. However, dogs did not match the behavior of the demonstrator (see Fugazza et al., 2019 for a discussion on this topic). These results raise important questions regarding what sort of mechanisms dogs are using in the test. From our data, dogs are not copying or imitating the human demonstrator. We suggest other socially biased learning mechanisms are being used, such as local enhancement, stimulus enhancement and/or social facilitation (Heyes and Galef, 1996). Social facilitation occurs when the presence of a demonstrator increases the chances of the observer to perform the same action, whilst stimulus/local enhancement (Spence, 1937; Thorpe, 1956) happens when there is an interaction between a demonstrator and an object or a place increasing the chances of the observer to interact with the same object or move towards the same place. These are two of the most common processes that ground socially biased learning (Hoppitt and Laland, 2013). Taken from the characteristics of our setting and our task, not finding significant results for matching, means that local and stimulus enhancement are possible. Even though we tend to consider the demonstrator or the movement the demonstrator does the most salient stimuli, it is possible that, for dogs, the baited bowl is in fact more salient. If that is the case, the individual might use their time during testing to try passing the fence to reach the food, without processing the detour itself. Thus, the dog could keep trying to transpass in any direction, eventually succeeding to get to the end of the fence and to the food. Therefore, we must consider that the local in which the demonstrator arrives (baited bowl) and the stimulus in the inner vertex of the fence (baited bowl) might be functioning as the driver for the dogs’ behavior. According to Heyes (1993) and other researchers such as Flynn and Whiten (2013), animals can learn the affordances of situations/contexts, in our case the V detour task, and emulate the outcome of another individual’s behavior. If dogs are using emulation in this test, they would achieve the consequence of the task, i.e., getting to the baited bowl, without reproducing the same behavior or behavioral sequence of the demonstrator. In fact, Mersmann et al. (2011) have tested the hypothesis that dogs use simpler mechanisms than imitation to solve social learning tasks. Part of their study was to investigate the underlying mechanisms of dogs’ performance in the V detour task and they argue that stimulus enhancement and affordance learning are powerful ways to solve this sort of problem for these animals. An interesting approach for further studies would be to look only at the subjects who completed the test within the same time as provided by pre-test, to deeper investigate the role of individual learning. Here, we have made a methodological choice to use a shorter duration at pre-test to control for habituation to the task while still providing enough time to solve the detour.

The analyses of the behavior of the dogs showed that success was lower if the dog looked at their owner at some point, when considering all trials, but not when analyzing first trials only. Possibly, in the cases when dogs looked at the owner, they did not know what to do and were trying to extract some information from their owner’s potential reaction (e.g., Merola et al., 2014). Moreover, when considering all trials, we found that the greater the persistence score, the distraction score and latency to reach the fence, the higher the time to solve the task, which is explained by logical time allocation by the subjects. On the other hand, the greater the persistence score, the distraction score, and time spent looking at the demonstrator during the demonstration of the test, the lower the latency to reach the fence. This was true for both all trials and first trials only with the exception of the distraction score that had an effect only when analyzing all trials, which, again, could be explained by greater interest in the baited bowl in the beginning of the test. However, latency to reach the fence was higher when the dog stayed besides their owner, a result of logical time allocation.

Interestingly, the emotional group (positive, negative, or neutral) had no effect on any of the four dependent variables (success, time to solve the task, latency to reach the fence and matching).

Dogs are known to be very good readers of human gestures, such as pointing and gazing (Cabral and Savalli, 2020), human body postures (Vas et al., 2005), and human facial expressions (Albuquerque et al., 2016, 2018; Correia-Caeiro et al., 2020). Furthermore, studies such as those from Turcsán et al. (2015) and Buttelmann and Tomasello (2013) show that the perception of human emotional expressions can differentially guide dogs’ behavior into choosing one of two objects and those of Merola et al. (2012, 2014) demonstrate that dogs obtain information from humans’ emotional reactions in order to interact – or not – with an unfamiliar object or situation. Not only emotional expressions but the attitude of a person towards the owner can channel how dogs behave and change their response depending on what they have observed (Chijiiwa et al., 2015). Moreover, there is evidence (Müller et al., 2015) that dogs trained with negative emotional expressions learned the contingencies of their task slower than dogs trained with positive emotional expressions. However, in our study, the valence (positive, negative or neutral) of the emotional display phase did not affect subjects’ performance.

Taking all the above into account, it is quite surprising that the emotional display our subjects witnessed in this study had no significant effect on their responses in this social learning task. One possible explanation is that the interval between the emotional display phase and the actual testing was too long and dogs did not remember what they have witnessed between the demonstrator and the owner. Fiset et al. (2003) studied dogs’ operational memory and described an above chance performance in an object permanence task for up to 240 s without distractors. In our case, the temporal space between the emotional display phase and the test not only included several distractions (due to a very rich environment) but also was over the mentioned interval, taking at least 5 min for the transference from one phase to the other. In fact, taking memory into account is not only important as is necessary, especially when studying social animals, who live in cohesive groups. We believe that further exploring this issue is critical to understanding the influence of emotional cues on the performance of dogs in social learning tasks. We know that dogs are capable of discriminating and recognizing human emotional expressions (e.g., Albuquerque et al., 2016). However, there is still a lack of evidence regarding for how long these animals can store this sort of information in their memory. In 2018, Proops and colleagues showed that horses can remember human facial expressions in such way that after seeing pictures of a face showing positive or negative emotion, they will respond differently when later they are presented to the real person. This study shows that non-human animals can indeed remember facial expressions of humans, or at least the sensation of seeing them angry or happy. However, they presented the emotional stimuli to the horses for a longer time compared to our study, which may have been critical for the storage of the emotional information, and they did not use food in their experimental design, which can function as an important distraction from the task.

Another possible explanation is that the used emotional displays were not salient enough to change the value of the demonstrator or to change the valence of the experimental setting itself, especially because there was food involved (see Chijiiwa et al., 2015 for a discussion on that). As discussed above, the baited bowl may have worked as the salient stimulus for the dogs, instead of the emotionally charged demonstrator and the demonstrator’s behavior during the task. At the same time, it is possible that the social learning task was too easy, thus diluting the relevance of the information and masking any potential effects of the emotional display. Further studies controlling for that are necessary. Finally, the presence of the owner during the emotional phase and the test may have caused an interference on how dogs perceived the human in terms of being more or less positive, more or less negative. According to Payne et al. (2015) the presence of a person can attenuate the effect of stressful events. In fact, the presence of the owner may function as a safe haven and may play a secure base effect.

Social learning is particularly effective among social animals (Fedurek et al., 2015) and dogs are one of the species that benefit from it (Range et al., 2009). Dogs are capable to obtain, store, and use information through demonstration of people as well as other dogs in observational and manipulative tasks (Range et al., 2009; 2007; Scandurra et al., 2016). At the same time, dogs can discriminate (Nagasawa et al., 2011), categorize (Müller et al., 2015), recognize (Albuquerque et al., 2016), and respond (Albuquerque et al., 2018) to emotional expressions, which allow them to assess the reactions, motivational states and intentions of others. In fact, the ability to perceive the emotions of others is one of the main social regulation mechanisms (Gross, 1998) and domestic dogs possess that as well. Here, we demonstrate that the mechanisms involved in observational social learning must be looked more in depth, since classical ideas of copying or imitation seem to not be likely from recent evidence. Most importantly, emotional cues did not interfere on our subjects’ performance, meaning that our hypothesis was not true, at least by using the sort of experimental procedures we have. The emotional display phase did not affect the learning environment, impairing or aiding social learning, or changing the value of the demonstrator and the information she was providing in a relevant way to decrease or increase the speed and the quality of learning. Even though our results do not corroborate the hypotheses raised by our team, they add important aspects to the literature and pave the way for further investigation in the dog cognition and social behavior areas.

## Data Availability Statement

The raw data supporting the conclusions of this article will be made available by the authors, without undue reservation.

## Ethics Statement

The animal study was reviewed and approved by the University of São Paulo Animal Ethics Committee. Written informed consent was obtained from the owners for the participation of their animals in this study. Written informed consent was obtained from the individual(s) for the publication of any potentially identifiable images or data included in this article.

## Author Contributions

NA and BR designed the experiment. NA collected the data. FC coded the videos. CS and NA analyzed the data. All authors wrote the manuscript and approved it for publication.

## Conflict of Interest

The authors declare that the research was conducted in the absence of any commercial or financial relationships that could be construed as a potential conflict of interest.

## Footnotes

1 www.solomoncoder.com
