# Supplementary material for: Do Emotional Cues Influence the Performance of Domestic Dogs in an Observational Learning Task?
Source: Front Psychol. 2021 May 20;12:615074. doi: 10.3389/fpsyg.2021.615074 (PMC8172801; doi:10.3389/fpsyg.2021.615074)
Supplement: Supplementary file 1 [file Table_1.docx]

**Table S1. Ethogram used for codification of the videos**

| **Category** | **Behavioral category** | **Description** |
| --- | --- | --- |
| **Interest 1** | Dig in front of the fence. | With their body turned towards the bowl, the dog, with their front paws, revolves the soil, removing soil or not and making a hole or not. |
|  | Touch the fence. | The dog touches the fence with any part of their body, standing within 10 cm away of the fence. The dog can be show any body position when touching the fence. |
|  | Sniff around the fence. | The dog sniffs the area around the fence, with his head directed towards the ground (with his snout close to the ground). |
| **Interest 2** | Look at the bowl. | The dog positions their head towards the baited bowl, standing within the marked area around the fence. At this point, the dog can also detect olfactive cues from the food. |
| **Interest 3** | Standing next to the owner. | The dog remains still within a 0.50m radius, with the owner as the center. |
| **Distraction** | Urinating / defecating. | The dog urinates / defecates. |
|  | Search for noises. | The dog moves their body following sounds from the environment. |
|  | Sniff the environment. | The dog sniffs the environment outside the marked area around the fence. |
|  | Dig the soil. | The dog revolves the soil, with their front legs, standing outside the marked area around the fence, removing soil or not, making a hole or not. |
| **Attention** | Look at the owner. | The dog turns their head towards the owner. |
|  | Look at the demonstrator. | The dog turns their head towards the demonstrator. |
